# Effects of growth years on ginsenoside biosynthesis of wild ginseng and cultivated ginseng

**DOI:** 10.1186/s12864-022-08570-0

**Published:** 2022-04-23

**Authors:** Xiaoxue Fang, Manqi Wang, Xinteng Zhou, Huan Wang, Huaying Wang, Hongxing Xiao

**Affiliations:** Key Laboratory of Molecular Epigenetics of Ministry of Education, Northeast Normal University, Changchun, 130024 China

**Keywords:** *Panax ginseng* c.a. Meyer, Transcriptome, Transcription factors, WGCNA

## Abstract

**Background:**

Ginsenoside, as the main active substance in ginseng, has the function of treating various diseases. However, the ginsenosides content of cultivated ginseng is obviously affected by the growth years, but the molecular mechanism is not clear. In addition, there are significant differences in morphology and physiology between wild ginseng and cultivated ginseng, and the effect of growth years on ginsenoside synthesis not yet understood in wild ginseng.

**Results:**

Transcriptome sequencing on the roots, stems and leaves of cultivated ginseng and wild ginseng with different growth years was performed in this study, exploring the effect of growth years on gene expression in ginseng. The number of differentially expressed genes (DEGs) from comparison groups in cultivated ginseng was higher than that in wild ginseng. The result of weighted gene co-expression network analysis (WGCNA) showed that growth years significantly affected the gene expression of Mitogen-activated protein kinases (MAPK) signaling pathway and terpenoid backbone biosynthesis pathway in cultivated ginseng, but had no effects in wild ginseng. Furthermore, the growth years had significant effects on the genes related to ginsenoside synthesis in cultivated ginseng, and the effects were different in the roots, stems and leaves. However, it had little influence on the expression of genes related to ginsenoside synthesis in wild ginseng. Growth years might affect the expression of genes for ginsenoside synthesis by influencing the expression of these transcription factors (TFs), like my elob lastosis (MYB), NAM, ATAF1 and 2, and CUC2 (NAC), APETALA2/ethylene-responsive factor (AP2/ERF), basic helix-loop-helix (bHLH) and WRKY, etc., thereby affecting the content of ginsenosides.

**Conclusions:**

This study complemented the gaps in the genetic information of wild ginseng in different growth periods and helped to clarify the potential mechanisms of the effect of growth years on the physiological state in wild ginseng and cultivated ginseng, which also provided a new insight into the mechanism of ginsenoside regulation.

**Supplementary Information:**

The online version contains supplementary material available at 10.1186/s12864-022-08570-0.

## Background

Ginseng (*Panax ginseng* C.A. Meyer) belongs to the genus *Panax* (Araliaceae family) and has been widely used as a significant source of natural medicine for thousands of years in East Asia, particularly in China, Korea and Japan [1]. Ginseng has been highly valued and vigorously promoted due to its important medicinal properties. The pharmacological active substances include ginsenosides, flavonoids and polysaccharides, etc., among which ginsenosides are the main bioactive compounds in ginseng [2]. Ginsenosides have various biological activities, such as immune modulation, anti-inflammation, anti-tumor, anti-amnestic and anti-aging activities [3–6]. In addition, ginsenosides also have a defensive effect on pathogenic microorganisms and herbivorous insects [7, 8].

Ginsenosides, triterpenoid saponins with a four-ring skeleton structure, are unique to ginseng genus. To date, more than 200 triterpene saponins have been isolated and characterized from the root, stem, leaf, flower and fruit of *P. ginseng* [9]. There are two main classes of ginsenosides based on the skeletons of their aglycones, namely, the dammarane type and the oleanane type. Dammarane type ginsenosides are divided into two groups based on their structure, the protopanaxadiol (PPD) group, including Ra1, Ra2, Rb1, Rb2, Rb3, Rc, Rd, Rg3, Rh2 and others and protopanaxatriol (PPT) group included Rg1, Rg2, Re, Rf, Rh1 and so on, while oleanane-group ginsenoside only has one saponin, Ro [10, 11]. Based on previous studies, the biosynthetic pathways of ginsenosides are as follows. Firstly, derivescisopentenyl pyrophosphate (IPP) and dimethylallyl pyrophosphate (DMAPP) are synthesized by the mevalonic acid (MVA) and 2-C-methyl-D-erythritol-4-phosphate (MEP) pathways, respectively. The MVA pathway is located in cytosol while the MEP pathway is in chloroplast. Then, 2,3-oxidosqualene is produced by IPP and DMAPP via cyclization reactions catalyzed by geranyl pyrophosphate synthase (GPS), farnesyl pyrophosphate synthase (FPS), squalene synthase (SS) and squalene epoxidase (SE) [12]. The 2, 3-oxidosqualene is cyclized by oxidosqualene cyclases (OSCs), generating the two triterpenoid backbones, dammarenediol-II and β-amyrin. The OSC enzyme family include dammarenediol synthase (DS) and β-Amyrin synthase (β-AS). Finally, triterpenoids are modified by some specific cytochrome P450 (CYP450) and UDP-glycosyltransferases (UGTs), resulting in a number of various ginsenosides [13].

The content of ginsenosides were various among different tissues of ginsengs and ginsengs with different growing years for ginsengs. Previous study had suggested the content of ginsenosides was different in various tissues, and it was significantly higher in roots than that in the stems and leaves of cultivated ginseng [14]. It was generally believed that the longer the cultivated ginseng grows, the higher the content of ginsenosides, which has been reported in many studies [15]. For example, the content of ginsenosides risen with the increase of cultivation years by comparing the content of ginsenosides from one to thirteen years of cultivated ginsengs [16]. Measurement of ginsenosides content from 1 to 6 years old cultivated ginseng by high-performance liquid chromatography (HPLC) indicated 5 and 6-year-old cultivated ginseng had high Ro/Re ratio, where that of cultivated ginseng at 2 and 3 years old was lower [17]. In addition, it has been reported that the content of total ginsenosides varied from 3.45% to 6.25% in wild ginseng grown for 1 to 18 years [18]. The molecular mechanism of the relationship between the genes related to ginsenoside synthesis and the growing years was not available in wild ginseng.

In order to reveal the molecular mechanism of ginsenosides content differences, transcriptome sequencing analysis was performed on cultivated ginseng. At present, transcriptome sequencing technology had made great progress in the complex biosynthetic pathway of ginsenosides and the expression of related genes. A total of 38 encoding enzymes were identified to be involved in ginsenoside biosynthesis pathway by transcriptome sequencing of 1-year-old and 6-year-old ginseng roots [19]. In different tissues of 5-year-old ginseng, the expression level of the MEP pathway genes were similar to those of the MVA pathway gens in roots, but higher in leaves [20]. Although some achievements had been made, it was not enough to fully understand the expression differences of genes related to ginsenoside biosynthesis in different tissues and growth years of cultivated ginseng. Furthermore, the above studies were based on cultivated ginseng, and it remained to be investigated whether the expression of genes related to ginsenoside synthesis in wild ginseng would be affected by growing years.

Ginsengs are mainly obtained through cultivation via modern agricultural technologies due to endangered resource of wild ginseng resulted from excessive consumption [21]. Cultivated ginseng is planted in farmland and grows fast. Generally, cultivated ginseng faces great pressure from various plant diseases and does not usually survive beyond six years, and yet, the growth period of cultivated ginseng also has reached 15 years [22]. In contrast, wild ginseng grows naturally in the forest, and the growth rate of wild ginseng is much slower than that of cultivated ginseng, then wild ginseng can grow up to hundreds of years under natural conditions [23–25]. Hence, in order to compare the effects of growth years on the ginsenoside biosynthesis in wild and cultivated ginsengs. The high-throughput transcriptome sequencing was performed on roots, stems and leaves of wild ginseng and cultivated ginseng with different growing years to explore the effect of growing years and tissues on gene expression, especially the genes involved in ginsenoside biosynthesis and regulation mechanism of ginsenoside biosynthesis. This study might provide information resources for improving transcriptome data of ginseng and also had profound significance for further research on ginseng cultivation breeding and related functional candidate genes. Identifying the effected of growing years on key enzyme genes in the ginsenoside biosynthetic pathway in wild and cultivated ginsengs would be an important step in improving ginsenoside production.

## Results

### Overview of RNA-seq data

In our study, we selected cultivated ginseng with a growth period of 1 year and 6 years from Jingyu county since cultivated ginseng generally grew for less than 6 years. The growth years of wild ginseng collected were 1 year and 20 years, respectively. In order to investigate the changes of gene expression related to ginsenoside synthesis in ginseng grown over 6 years, we also collected cultivated ginseng grown for 6 years and 15 years from Taishang town. A total of 39 individuals were sequenced with an average amount of clean bases per individual was 6.5 Gb. After filtering low-quality reads, the clean reads were mapped to the ginseng reference genome with an average mapping rate of 87.44%. The gene expression variations in biological replicates in each sample were evaluated, and the correlation values were high between them (Additional file 1: Fig. S1, r^2^ > 0.80), except for between the two replicates in TSBT_R (Additional file 1: Fig. S1, r^2^ = 0.56). However, considering that only two samples in the TSBT_R and the r^2^ > 0.50. Therefore, all samples were used for subsequent analysis.

### Differential expression analysis in three groups 

**Fig. 1:**
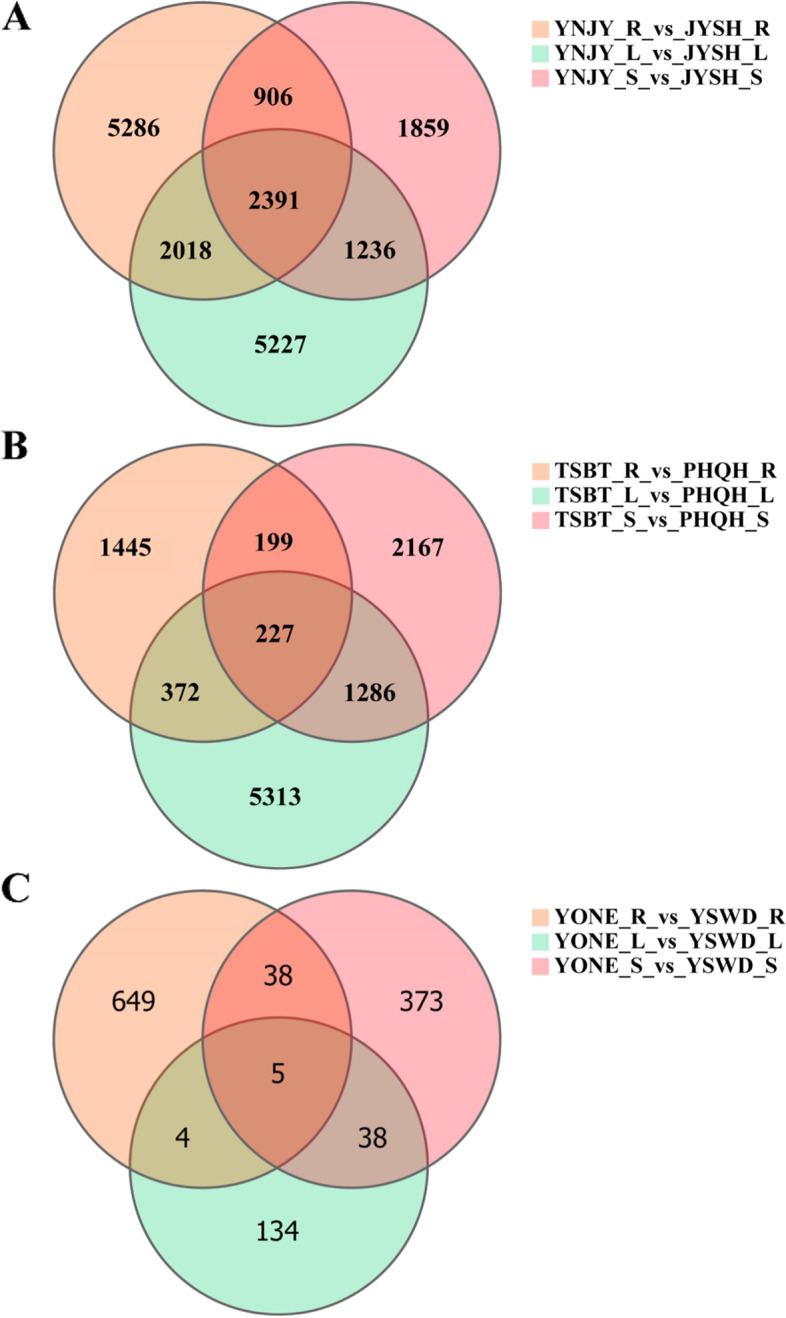
Venn diagram of differentially expressed genes (DEGs) in different compared groups. The overlapping portions of the different circles represent the number of DEGs common to these groups. **A** YNJY_vs_JYSH; **B** TSBT_vs_PHQH; **C** YONE_vs_YSWD. R, root; L, leaf; S, stem

The all ginsengs were divided into three comparison groups, including cultivated ginseng YNJY_vs_JYSH and TSBT_vs_PHQH groups, and wild ginseng YONE_vs_YSWD group. There were 18,923, 11,009 and 1,241 DEGs in YNJY_vs_JYSH, TSBT_vs_PHQH and YONE_vs_YSWD, respectively (Fig. 1). The number of differentially expressed genes (DEGs) in cultivated ginseng with different growth years was higher than that in wild ginseng with different growth years. Furthermore, the number of DEGs in YNJY_vs_JYSH was larger than that in TSBT_vs_PHQH. In addition, the numbers of DEGs in different tissues of three comparison groups were also different. In YNJY_vs_JYSH, the number of DEGs in roots were almost the same as that in leaves, while DEGs in stems were the least (6,392) (Fig. 1A). In TSBT_vs_PHQH, the number of DEGs in leaves was the highest (7,198), while DEGs in roots were the least (2,243) (Fig. 1B). In YONE_vs_YSWD, the number of DEGs in root was the highest (696), then DEGs in leaves were the least (181) (Fig. 1C).

### Construction of gene co-expression network

Weighted gene co-expression network (WGCNA) was constructed using 39,086, 38,823, and 39,780 genes from YNJY_vs_JYSH, TSBT_vs_PHQH and YONE_vs_YSWD, and the soft thresholding of three groups were set at 14, 10 and 8, respectively (Additional file 1: Fig. S2). While the scale-free topology fit index reached 0.85, indicating approximate scale-free topology (Additional file 1: Fig. S2). Finally, there were 34, 57 and 69 distinct modules were identified in YNJY_vs_JYSH, TSBT_vs_PHQH, YONE_vs_YSWD, respectively (Additional file 1: Fig. S3).

**Fig. 2:**
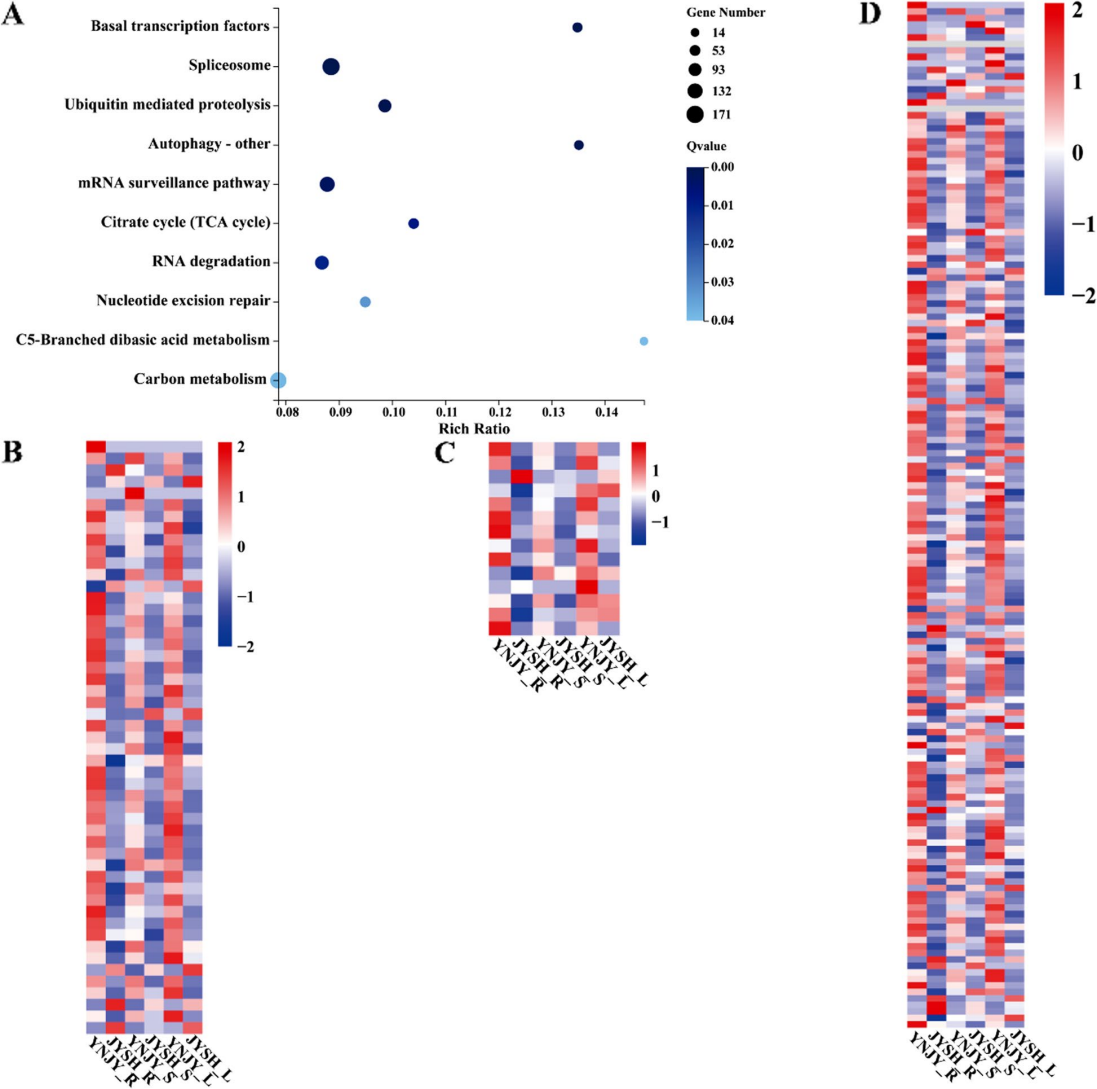
Kyoto Encyclopedia of Genes and Genomes (KEGG) enrichment analysis and expression profile associated with the blue module in YNJY_vs_JYSH group. **A**, KEGG pathway enrichment of blue module; **B**, expression of genes related to Citrate cycle (TCA cycle); **C**, expression of genes related to C5-Branched dibasic acid metabolism; **D**, expression of genes related to Carbon metabolism. R, root; L, leaf; S, stem. Various color blocks represent FPKM (Fragments Per Kilobase of transcript per Million mapped reads) normalized expression values. Red represents high gene expression, and blue represents low gene expression

**Fig. 3:**
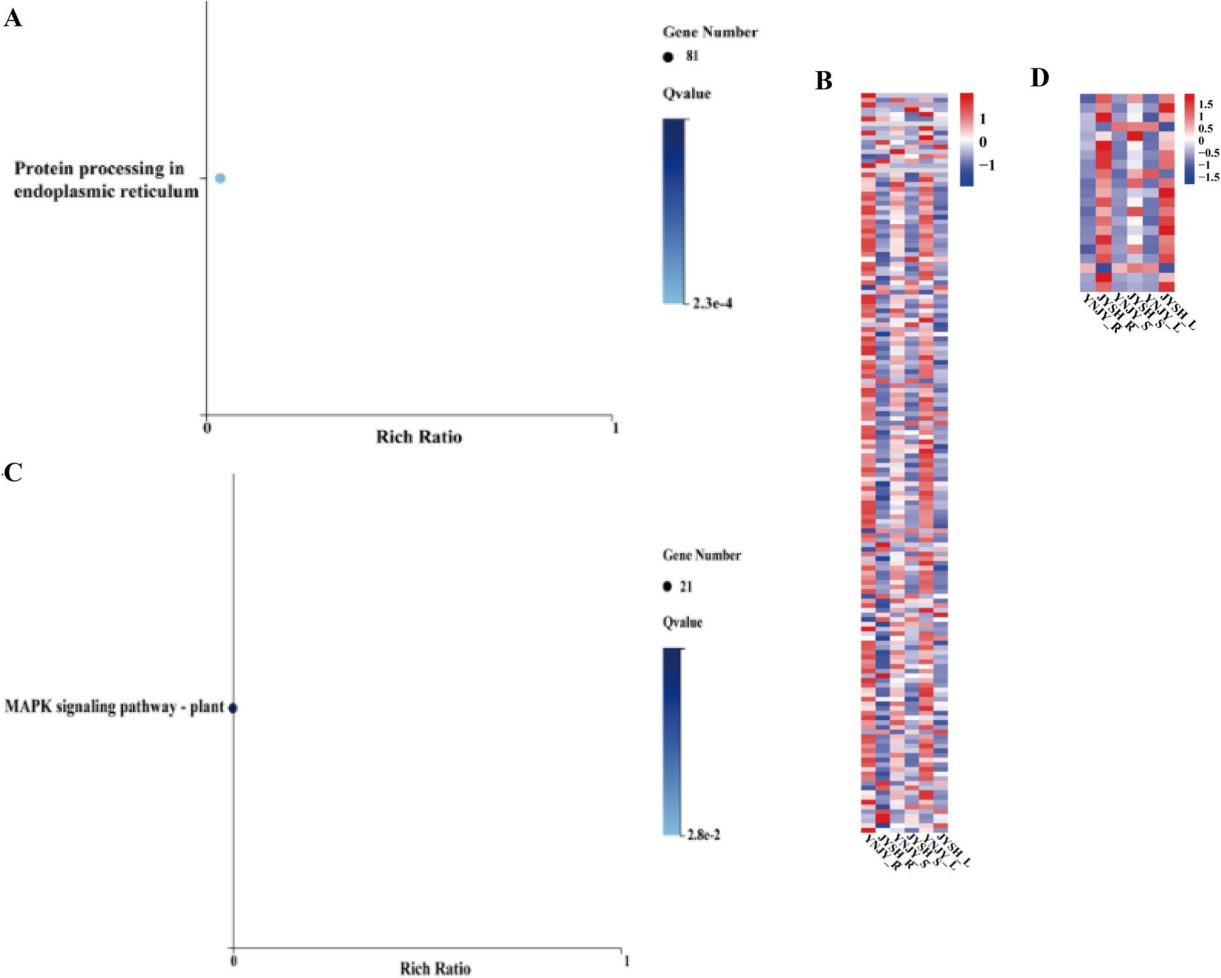
Kyoto Encyclopedia of Genes and Genomes (KEGG) enrichment analysis and expression profile associated with the red module and royalblue module in YNJY_vs_JYSH group. **A**, KEGG pathway enrichment of red module; **B**, expression of genes related to Protein processing in endoplasmic reticulum; **C**, KEGG pathway enrichment of royalblue module; **D**, expression of genes related to MAPK signaling pathway-plant. R, root; L, leaf; S, stem. Various color blocks represent FPKM (Fragments Per Kilobase of transcript per Million mapped reads) normalized expression values. Red represents high gene expression, and blue represents low gene expression

In WGCNA, the module eigengene (ME) was representative of the corresponding module’s gene expression profile correlated with a defined trait (growing years). Several modules were significantly correlated with the growing years (*p* < 0.05). In YNJY_vs_JYSH, blue, royalblue and red modules were significantly correlated with growing years, and the correlation coefficient were 0.91, 0.90 and -0.96, respectively (*p* < 0.05) (Additional file 1: Fig. S3). In blue module, these genes were enriched in the citric acid cycle (TCA cycle) (ko00020), c5-branched secondary acid metabolism (ko00660) and carbon metabolism (ko01200) (Fig. 2A). And the most genes in these pathways were highly expressed in YNJY (Fig. 2B, C, D). In red module, genes were enriched in protein processing in endoplasmic reticulum (ko04141) (Fig. 3A). Differential clustering of genes involved in this pathway showed that most genes were expressed at higher levels in YNJY than in JYSH (Fig. 3B). In royalblue module, the pathway enriched for these genes was Mitogen-activated protein kinases (MAPK) signaling pathway-plant (ko04016), in which genes were up-regulated in JYSH (Fig. 3C, D).

**Fig. 4:**
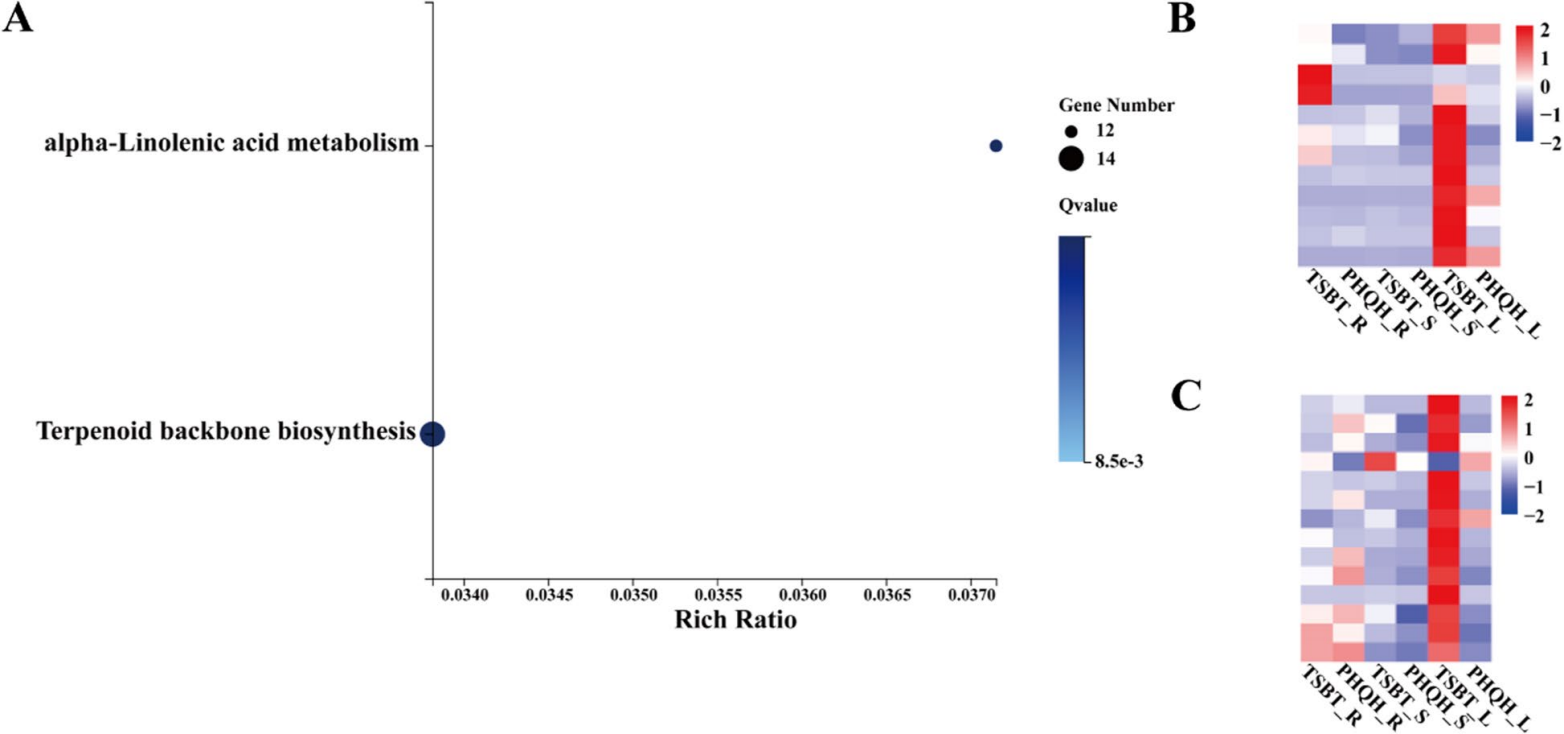
Kyoto Encyclopedia of Genes and Genomes (KEGG) enrichment and differential clustering analysis of the grey60 module in TSBT_vs_PHQH group. **A**, KEGG pathway enrichment of grey60 module; **B**, expression of genes related to alpha-Linolenic acid; **C**, expression of genes related to terpenoid backbone biosynthesis. R, root; L, leaf; S, stem. Various color blocks represent FPKM (Fragments Per Kilobase of transcript per Million mapped reads) normalized expression values. Red represents high gene expression, and blue represents low gene expression

In TSBT_vs_PHQH, three modules were significantly correlated with growing years, namely white, grey60 and skyblue3 modules (*p* < 0.05). However, only Alpha-linolenic acid metabolism (ko00592) and terpenoid backbone biosynthesis (ko00900) were enriched in grey60 module (Fig. 4A), and the expression levels of most genes of these pathways were lower in PHQH than that in TSBT (Fig. 4B, C). Nevertheless, there were no enriched pathways in the significant related modules (orangered4 and salmon4 modules) for YONE_vs_YSWD.

In addition, a large number of genes for ginsenoside biosynthesis were found in four modules related to growth years, such as FPS, diphosphomevalonate decarboxylase (MVD), phosphomevalonate kinase (PMK), UGTs, SS, SE and CYP450 were found in the blue and royalblue modules from YNJY_vs_JYSH. In the grey60 module from TSBT_vs_PHQH, there were 10 enzymes involved in the synthesis of ginsenosides were identified, including hydroxymethylglutaryl-CoA reductase (HMGR), (E)-4-hydroxy-3-methylbut-2-enyl-diphosphate synthase (ISPG), 4-hydroxy-3-methylbut-2-en-1-yl diphosphate reductase (ISPH), SS, 1-deoxy-D-xylulose-5-phosphate synthase (DXS), DS, 2-C-methyl-D-erythritol 2,4-cyclodiphosphate synthase (ISPF), acetyl-CoA C-acetyltransferase (AACT) and hydroxymethylglutaryl-CoA synthase (HMGS). There was only 1 enzyme involved in ginsenoside biosynthesis in the orangered4 module from YONE_vs_YSWD, which was 4-diphosphocytidyl-2-C-methyl-D-erythritol kinase (ISPE) (Table 1).

**Table 1:** Genes involved in the ginsenoside biosynthetic pathway in the modules

Module	Gene_id	Gene family
blue	Pg_S0304.36	FPS
	Pg_S3074.4	MVD
	Pg_S3321.6	PMK
	Pg_S6708.3	UGTs
royalblue	Pg_S0992.8	SS
	Pg_S2390.5	UGTs
	Pg_S3064.5	SE
	Pg_S3293.6	CYP450
	Pg_S4157.4	UGTs
	Pg_S4174.7	UGTs
	Pg_S4493.1	UGTs
	Pg_S4733.5	CYP450
	Pg_S6308.10	SE
grey60	Pg_S0126.10	HMGR
	Pg_S0247.51	IspG
	Pg_S0913.16	HMGR
	Pg_S1005.15	IspH
	Pg_S1678.33	SS
	Pg_S1908.21	DXS
	Pg_S3318.3	DS
	Pg_S4604.8	IspF
	Pg_S6240.3	AACT
	Pg_S6896.2	HMGS
orangered4	Pg_S2198.2	IspE

**Fig. 5:**
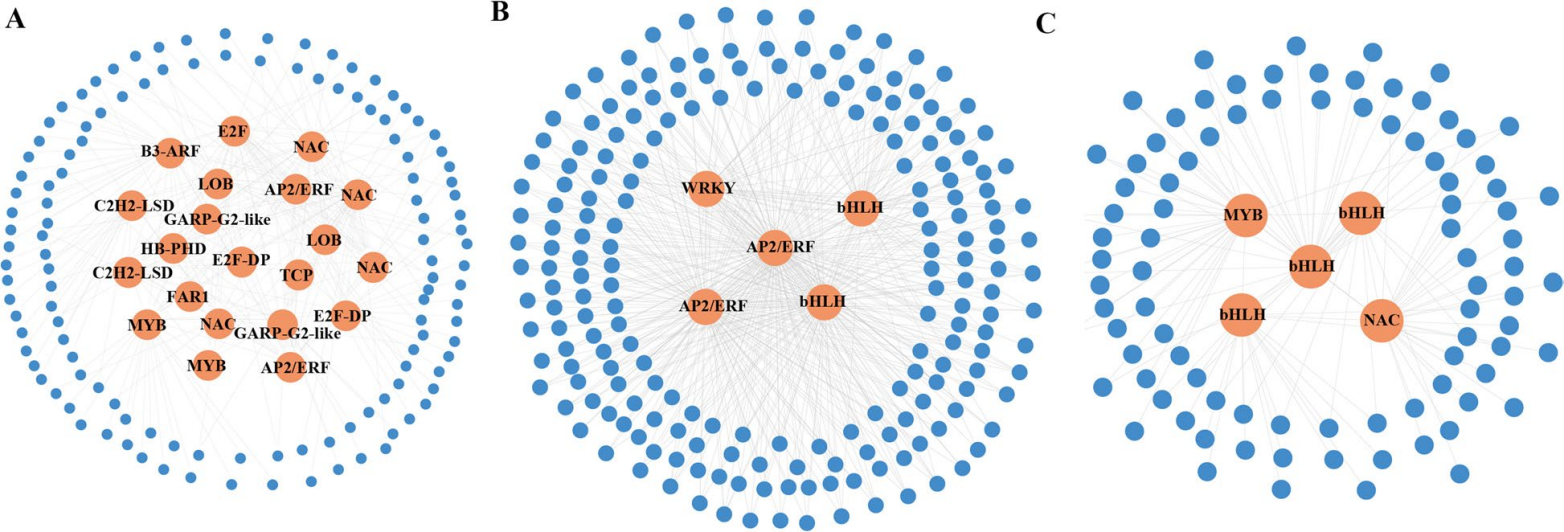
Gene co-expression subnetwork in the **A** blue, **B** royalblue, **C** grey60 modules

**Fig. 6:**
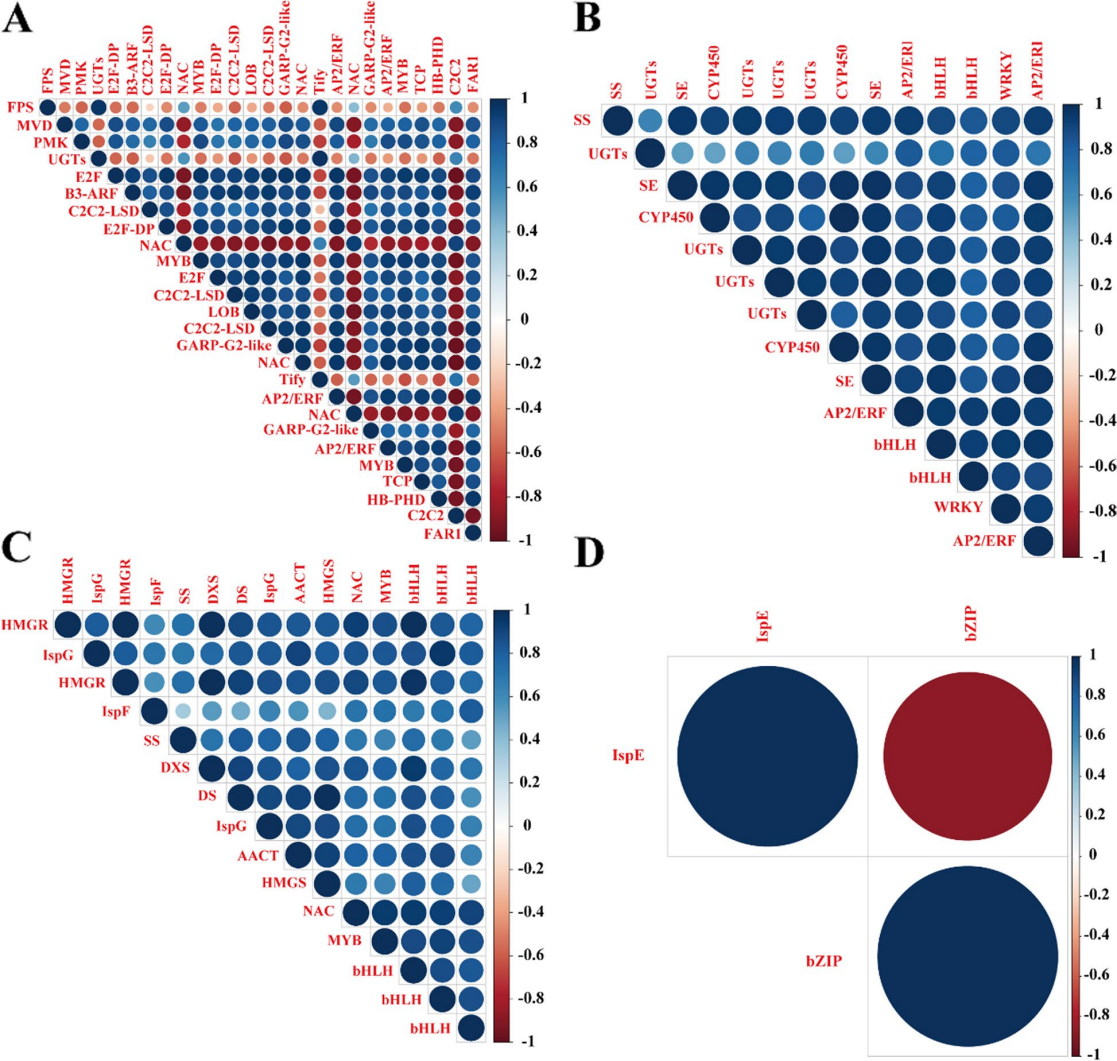
Correlation analysis of transcription factors (TFs) and polysaccharide synthesis genes in the (**A**) blue, (B) royalblue, (**C**) grey60 and (**D**) orangered4 modules. Network was reconstructed by edge weight cutoff = 0.25 and visualized by Cytoscape

In order to find the key regulatory transcription factors (TFs) related to ginsenoside biosynthesis from these modules, a gene association network was constructed for each module. There were 12 TFs were identified in blue module, including B3-Auxin Response Factor (B3-ARF) (Pg_S0055.1), E2F (Pg_S0029.9, Pg_S1183.4, Pg_S0517.8 and Pg_S0284.10), C2C2-lesion simulating disease (C2C2-LSD) (Pg_S1482.2 and Pg_S5995.1), NAM, ATAF1 and 2, and CUC2 (NAC) (Pg_S2339.2, Pg_S2698.4 and Pg_S4475.1), GARP-G2-like (Pg_S2430.1 and Pg_S4768.11), my elob lastosis (MYB) (Pg_S0682.20 and Pg_S5278.7), APETALA2/ethylene-responsive factor (AP2/ERF) (Pg_S4268.4 and Pg_S4859.1), LOB (Pg_S1960.28 and Pg_S0146.8), HB-PHD (Pg_S5577.18), FAR1(Pg_S7424.8) and TCP (Pg_S5350.8). The expression levels of these TFs were highly positively correlated with the expression levels of MVD and PMK. However, only the expression levels of Tify (Pg_S3560.17) was highly positively correlated with the expression levels of FPS and UGTs (Figs. 5A, 6A). There were 5 genes encoding 3 TFs were found in the royalblue module, including AP2/ERF (Pg_S0575.7 and Pg_S0315.1), basic helix-loop-helix (bHLH) (Pg_S1163.1 and Pg_S3713.24) and WRKY (Pg_S5167.2). Furthermore, the expression levels of AP2/ERF, bHLH and WRKY were highly related to the expression levels of UGTs, SS, SE and CYP450 (Figs. 5B, 6B). In the grey60 module, the expression of NAC (Pg_S1059.27), MYB (Pg_S1913.1) and bHLH (Pg_S4358.2, Pg_S6447.1 and Pg_S3268.1) were highly positively correlated with the expression levels of IspG, HMGR, IspH, SS, DXS, DS, IspF, AACT and HMGS (Figs. 5C, 6C). In the orangered4 module, only one TF for bZIP was found, and the expression levels of bZIP (Pg_S0602.25) was highly positively related to the expression levels of IspE (Fig. 6D). These results indicated that TFs might affect the expression of genes for ginsenoside synthesis.

### Identification of DEG related to ginsenoside biosynthesis

**Fig. 7:**
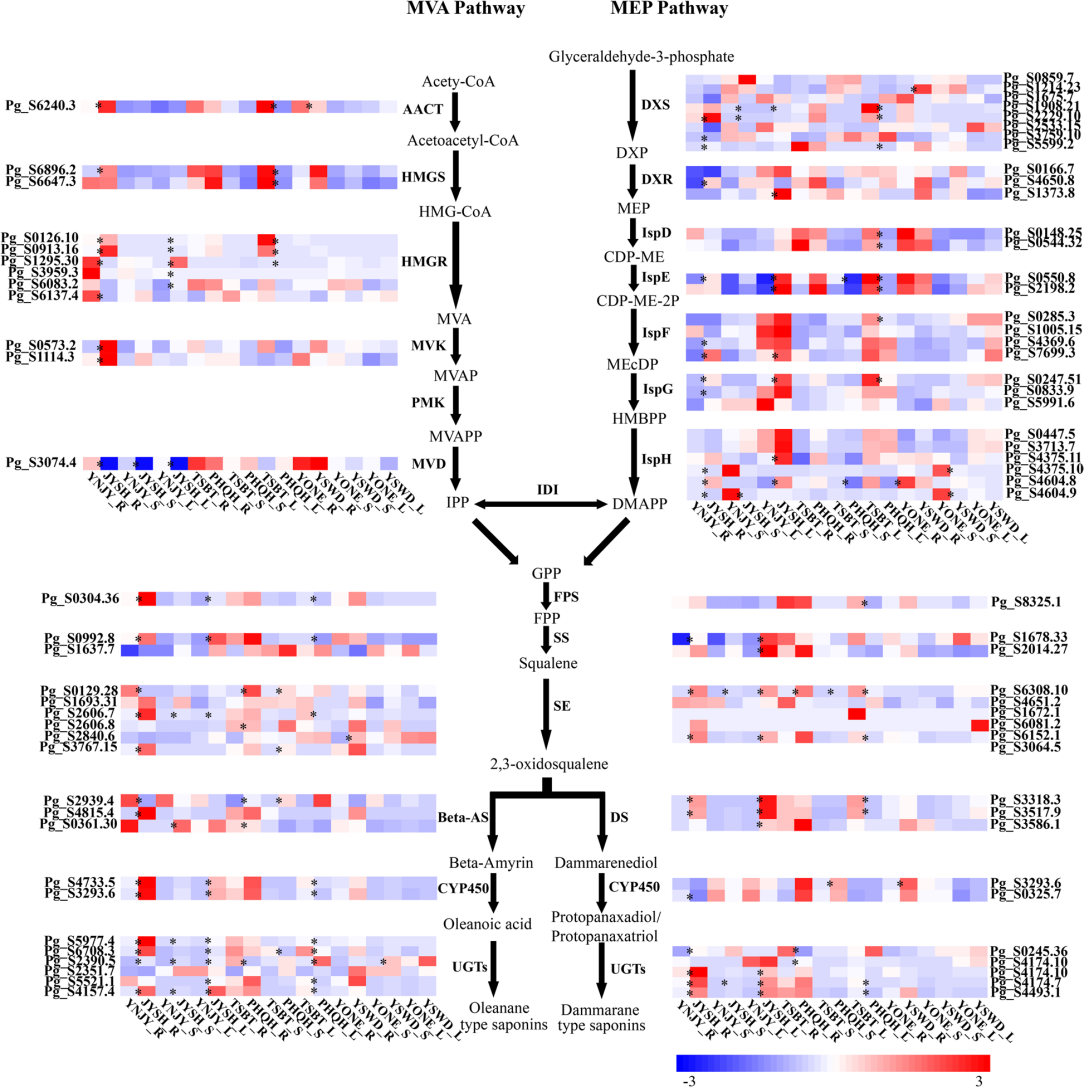
Heatmaps of DEG related to ginsenoside biosynthesis in YNJY_vs_JYSH, TSBT_vs_PHQH and YONE_vs _YSWD groups. * represent DEGs in each comparison group. Each row corresponds to a gene related to ginsenoside biosynthesis. R, root; L, leaf; S, stem. Various color blocks represent FPKM (Fragments Per Kilobase of transcript per Million mapped reads) normalized expression values. Red represents high gene expression, and blue represents low gene expression. *DMAPP* Dimethylallyl pyrophosphate, *MVA* Mevalonic acid, *MEP* 2-C-methyl-D-erythritol-4-phosphate, *GPS* Geranyl pyrophosphate synthase, *FPS* Farnesyl pyrophosphate synthase, *SS* Squalene synthase, *SE* Squalene epoxidase, *OSCs* Oxidosqualene cyclases, *DS* Dammarenediol synthase, *β-AS* β-Amyrin synthase, *CYP450s* Cytochrome P450s, *UGTs* UDP-glycosyltransferases, *ME* Module eigengene, *WGCNA* Weighted gene co-expression network analysis, *FPS* farnesyl diphosphate synthase, *MVD* Diphosphomevalonate decarboxylase, *PMK* Phosphomevalonate kinase, *HMGR* Hydroxymethylglutaryl-CoA reductase, *ISPG* (E)-4-hydroxy-3-methylbut-2-enyl-diphosphate synthase, *ISPH* 4-hydroxy-3-methylbut-2-en-1-yl diphosphate reductase, *DXS* 1-deoxy-D-xylulose-5-phosphate synthase *ISPF* 2-C-methyl-D-erythritol 2,4-cyclodiphosphate synthase, *AACT* Acetyl-CoA C-acetyltransferase, *HMGS* Hydroxymethylglutaryl-CoA synthase, *ISPE* 4-diphosphocytidyl-2-C-methyl-D-erythritol kinase, *MVK* Mevalonate Kinase, *DXR* 1-deoxy-D-xylulose-5-phosphate reductoisomerase

The expression level of genes in the ginsenoside biosynthesis pathway were further inspected among different tissues and sample of ginsengs. These genes were significantly various among cultivated ginseng with different growth years. In YNJY_vs_JYSH, most DEGs involved in the ginsenoside biosynthetic pathway showed higher expression levels in JYSH than in YNJY. Only genes encoded MVD was highly expressed in three tissues of YNJY. The numbers of DEGs involved in the ginsenoside biosynthesis pathway were in roots and leaves more than in stems (Fig. 7). Fourteen enzymes, including AACT, HMGS, Mevalonate Kinase (MVK), DXS, 1-deoxy-D-xylulose-5-phosphate reductoisomerase (DXR), ISPE, ISPF, ISPG, FPS, SS, SE, DS, CYP450 and UGTs were highly expressed in the roots of JYSH. In the stems, level of gene expression encoding the six enzymes (MVD, DXS, ISPH, SE, β-AS and UGTs) were significantly different, moreover, three (SE, β-AS and UGTs) of them were highly expressed in JYSH. In addition, the twelve enzymes included HMGR, DXR, ISPE, ISPF, ISPG, ISPH, FPS, SS, SE, DS, CYP450 and UGTs were highly expressed in leaves of JYSH (Fig. 7).

In TSBT_vs_PHQH, the numbers of DEGs associated with ginsenoside biosynthesis in leaves were significant more than that in roots and stems. In roots and stems, the expression level of six enzymes (ISPE, ISPH, SE, β-As, CYP450 and UGTs) were different, only SE and CYP450 showed higher expression levels in PHTS than that in TSBT, while the other DEGs were highly expressed in TSBT, including ISPE, ISPH, β-As and UGTs. In the leaves, sixteen enzymes (AACT, HMGS, HMGR, DXS, ISPD, ISPE, ISPF, ISPG, ISPH, FPS, SS, SE, β-As, DS, CYP450 and UGTs) were highly expressed in TSBT than in PHQH (Fig. 7).

In contrast, there were fewer DEGs for ginsenoside biosynthesis in the roots, stems and leaves of wild ginseng group (YONE_vs_YSWD) compared with cultivated ginseng comparison groups. Five enzymes, HMGS, DXS, SE, CYP450 and UGTs were highly expressed in the root of YSWD, however, only ISPH was highly expressed in the stem of YONE (Fig. 7). The result indicated that the growth years had little effect on ginsenoside synthesis in wild ginseng.

### Validation of DEGs by qRT-PCR

To ensure the reliability and accuracy of the results, fifteen DEGs of different ginseng tissue samples were randomly selected to verify the relative expression levels by qRT-PCR analysis. The validation results showed that the expression trends of examined genes were consistent with that derived from RNA-seq (Additional file 1: Fig. S4). The correlation between RNA-seq results and qRT-PCR experimental results was significant and the correlation coefficient was 0.864 by spearman test (*p* < 0.01).

## Discussion

### Expression profiles of the DEGs in different growth years of cultivated ginseng

Ginseng is a kind of perennial herbaceous plant, its morphology and physiology changes greatly during the growth process. Dynamic gene expression profiles of cultivated and wild ginsengs with different growth years were described. In the WGCNA of YNJY_vs_JYSH, the genes related to growth years were enriched in citrate cycle (TCA cycle) (ko00020), C5-Branched dibasic acid metabolism (ko00660) and Carbon metabolism (ko01200) in blue module, and the most genes from these pathways were highly expressed in YNJY (Fig. 2). These pathways were common primary metabolic pathways in plants. This might be that the primary metabolic pathway of one-year-old cultivated ginseng (YNJY) was more active. The morphological and physiological components of cultivated ginseng were basically mature after 6 years of growth, which might enhance its secondary metabolism, so primary metabolism decreased. This phenomenon also occurred in *Atractylodes atractylodes*. By comparing *A. atractylodes* with different growth years, suggesting that the productivity of secondary metabolism of *A. atractylodes* increased with cultivation years, possibly because it was used to resist the pressure in old plants [26].

Furthermore, the genes related to growth years were enriched in MAPK signaling pathway – plant in royalblue module from YNJY_vs_JYSH (Fig. 3C, D). This pathway is involved in plant disease resistance and abiotic stress response [27–29]. In *Arabidopsis thaliana*, overexpression of MKK2 gene increased MPK4 and MPK6 activities, thus enhancing plant frost resistance and salt tolerance [30]. Moreover, the most genes of this pathway were expressed a higher level in JYSH, indicating that six-years-old ginseng (JYSH) might have higher stress resistance. With the increase of growing years, cultivated ginseng faced more diseases and huge environmental pressure [21]. Hence, the expression of genes related to stress resistance pathways was continuously increased to resist the bad environment [31].

In the WGCNA of TSBT_vs_PHQH, the genes related to the terpenoid backbone biosynthesis were found in grey60 module (Fig. 4A). And the expression level of most genes in terpenoid backbone biosynthesis in TSBT was higher than in PHQH (Fig. 4C). Terpenoid backbone biosynthesis is a key step in the synthesis of ginsenosides for ginseng [32]. Previous studies had reported 15 years represented a turning point in the maturation of ginseng growth and the accumulation of these bioactive components in ginseng, moreover, most ginsenosides accumulated during years 5–15 and decreased after 15 years [33]. This might be related to the low expression of terpenoid backbone biosynthesis genes in ginseng that had been grown for more than 15 years (PHQH), resulting low ginsenosides content.

### Effects of growth years on ginsenosides biosynthesis in cultivated ginseng

As the main active ingredient in ginseng, ginsenosides have been used in the treatment of various diseases [13]. The aim of this study was to investigate the effects of growing years on genes related to ginsenoside biosynthesis in cultivated and wild ginsengs. In YNJY_vs_JYSH, most genes involved in the ginsenoside biosynthetic pathway showed higher expression levels in JYSH than in YNJY (Fig. 7). Previous studies have suggested that the contents of ginsenosides increased with cultivation ages in cultivated ginseng [17, 34]. The high expression level of these DEGs might contribute to the accumulation of ginsenosides in six-year-old cultivated ginseng (JYSH). Moreover, the amounts of secondary metabolites produced by plants for defense purposes increased under environmental stress conditions such as nutrient deficiency [35, 36]. Currently, ginsenosides, as secondary metabolites, have been shown to play an important physiological role to protect plants from pathogens [37]. When ginseng grown to six years old, it faced greater environmental pressure. Therefore, it was more necessary to synthesize ginsenoside to cope with many biotic or abiotic stresses [38, 39].

Interestingly, the number of DEGs involved in the ginsenoside biosynthetic pathway in roots and leaves were more than in stems for YNJY_vs_JYSH (Fig. 7). Furthermore, the number of DEGs related to ginsenoside biosynthesis in leaves was more than in stems and roots from TSBT_vs_PHQH (Fig. 7). The different expression profiles of genes related to ginsenoside synthesis in ginseng roots, stems and leaves suggested that there might be significant differences in ginsenosides or other triterpenoid precursors synthesized in different tissues of ginseng. More recently, the genes related to ginsenoside synthesis pathway were expressed in living cells, but not in vascular and xylem cells of ginseng by transcriptome sequencing analysis of different tissues of 5-year-old ginseng [20]. Moreover, the roots and stems are more lignified than the leaves. In addition, the ginsenosides were isolated and identified from different tissues of ginseng showed the main ginsenosides in root and leaf were Rb1, Rb2, Rc, Rd (PPD-type) and Re, Rg1, Rf (PPT-type), respectively [37]. The contents of ginsenosides Rg1, Re, Rf, Rg2, Rb1, Rc, Rb2 and Rd in periderm were significantly higher than those in middle column by HPLC [40]. This differentiation of gene expression probably leaded to the accumulation of different types of ginsenosides in the roots, stems and leaves, thus explaining tissue-specific ginsenosides production.

### Effects of growth years on ginsenosides biosynthesis in wild ginseng

The germplasm resources of wild ginseng are scarce, so there are few studies on gene expression level of wild ginseng. In our study, the numbers of DEGs in YNJY_vs_JYSH and TSBT_vs_PHQH was more than that in YONE_vs_YSWD, suggesting gene expression levels were less affected by growth years in wild ginseng compared with cultivated ginseng (Fig. 1). Wild ginseng grows more slowly than cultivated ginseng and can be grown in nature for hundreds of years, so it may be less affected by a few short decades [41]. Furthermore, genes related to ginsenoside biosynthesis, including HMGS, DXS and SE were highly expressed in the roots in YSWD than in YONE (Fig. 7). HMGS is a key catalytic enzyme in MVA pathway, and HMGS overexpression increases triterpenoids in *Ganoderma lucidum* [42]. The enhanced expression level of SE genes might cause increased production of triterpenoids in *Ganoderma lucidum* [43]. In addition, DXS is important in terpenoid biosynthesis, and DXS is the first rate-limiting enzyme of the MEP pathway [44]. However, there was no DEGs involved in ginsenoside biosynthesis in the leaves of YONE_vs_YSWD, indicating that the effect of growth years on ginsenoside biosynthesis in wild ginseng was mainly in roots, but had little effect on leaves (Fig. 7). Wild ginseng increases the number of leaves every 3–5 years, eventually producing 4–6 compound leaves. These leaves are then all shed and grow again from the first leaf [45]. The leaf state of twenty-year-old wild ginsengs was similar to that of one-year-old wild ginsengs, thus, the number of DEGs in the leaves was the least and there were few DEGs related to ginsenoside biosynthesis.

### TFs regulated gene network for ginsenoside biosynthesis

Biosynthesis of particular compounds is influenced by a variety of factors by influencing transcription factors (TFs), which regulate the expression of relevant genes [46, 47]. The genes associated with ginsenoside biosynthesis and TFs were found in four modules (blue, royalblue, grey60 and orangered4) by screening growth age-related modules using WGCNA in this study. In these modules, the expression level of MYB (Pg_S0682.20, Pg_S5278.7, Pg_S0682.20, Pg_S5278.7 and Pg_S1913.1), NAC (Pg_S2339.2, Pg_S2698.4, Pg_S1059.27 and Pg_S4475.1), AP2/ERF (Pg_S4268.4, Pg_S4859.1, Pg_S0575.7 and Pg_S0315.1), bHLH (Pg_S1163.1, Pg_S4358.2, Pg_S6447.1, Pg_S3268.1, Pg_S3713.24, Pg_S4358.2, Pg_S6447.1 and Pg_S3268.1), bZIP (Pg_S0602.25) and WRKY (Pg_S5167.2) were highly correlated with the expression level of genes related to ginsenoside biosynthesis (Fig. 6).

MYB family is the most widely distributed and powerful transcription factor [48]. PgMYB2 in ginseng, was confirmed to positively regulate the expression of DS in ginseng [49]. In our study, the expression level of MYB was also positively correlated with the expression of DS. In addition, the expression level of NAC and AP2/ERF highly correlated with the expression level of genes related to ginsenoside biosynthesis, such as HMGR, IspG, IspH, SS, DXS, DS, IspF, AACT and HMGS et al. In *Cyclocarya Paliurus* (Bata1) Iljinskaja, NAC might be involved in the synthesis of triterpenoids [50]. The expression levels of DS and SS and total saponins content in PnERF1 transgenic *P. notoginseng* cell lines were higher than those in non-transgenic cell lines [51]. Moreover, the expression of WRKY was highly related to the expression of SS, SE, CYP450 and UGTS. WRKY mainly exist in plants and play important roles in many biological processes [52]. PqWRKY1 isolated from *P. quinquefolius* enhanced the content of saponins by regulating the transcription of some genes related to biosynthesis of saponins [53]. There were 8 genes encoded bHLH, and expression levels of them were significant positive correlation with the expression of genes related to ginsenoside biosynthesis, such as UGTs, CYP450, SS, DS, IspF, AACT and SE et al. In *P. notoginseng*, the overexpression of tf PnbHLH caused the increase of saponins [54]. In YONE_vs_YSWD, only the expression of bZIP was highly positively related to the expression of IspE in orangered4 module. However, there were few studies on the effect of bZIP on the synthesis of ginsenosides in *Panax* genus, which required subsequent yeast one-hybrid or two-hybrid to verify its function. These results suggested These transcription factors play an important role in the bioregulatory synthesis of ginsenosides.

## Conclusions

A comprehensive transcriptome analysis was performed to investigate the effects of growing years on wild and cultivated ginseng, indicating the number of DEGs related to ginsenoside synthesis was affected by growth years in cultivated ginseng, and the degree of influence varies in different tissues. However, the DEGs associated with ginsenoside synthesis were little influenced in wild ginseng and no effect on leaves. In addition, MYB, NAC, AP2/ERF, bHLH, bZIP and WRKY were identified in modules associated with growth years, and the expression levels of these TFs were highly correlated with the expression level of HMGR, SS, DXS, DS, IspF, AACT and HMGS. These TFs might affect the content of ginsenosides by regulating the expression of genes for ginsenoside synthesis. These findings provided genetic resources and theoretical basis for further investigation of gene function in wild ginseng and cultivated ginseng, and also supplied new insights for understanding the metabolic regulation of ginsenosides in wild ginseng by growth years. It also provided scientific reference for understanding terpenoid biosynthesis of other cultivated and wild species of *Panax* genus.In future studies, wild ginseng and cultivated ginseng materials with different growth years will be collected and the ginsenosides content will be measured, and then combined transcriptome and metabolome analysis to further explore the effect of growth years on the ginsenosides synthesis mechanism in wild ginseng and cultivated ginseng [45].

## Methods

### Plant materials and preparation

One- and six-year-old cultivated ginsengs were collected from Jingyu county, Jilin province of China (marked as YNJY and JYSH, respectively), and the one- and twenty-year-old wild ginsengs were collected from Korean Autonomous County of Changbai, Jilin province (marked as YONE and YSWD, respectively). In addition, six-year-old cultivated ginsengs from Taishang town of Jilin province (marked as TSBT) were sampled, meanwhile, three samples of more than fifteen-year-old cultivated ginsengs from the same location were also collected (marked as PHQH) (Additional file 1: Table S1).

All cultivated ginseng and wild ginseng samples were collected in August 2018. After cleaning with distilled water on the spot, the roots, stems and leaves of all ginseng were separately cut into small pieces quickly and packaged in aluminum foils. Then these samples are immediately frozen in the liquid nitrogen and stored at -80℃. According to sampling location, these ginsengs were divided into three comparison groups, namely YNJY_vs_JYSH, TSBT_vs_PHQH and YONE_vs_YSWD.

### RNA extraction, cDNA library construction and sequencing

Total RNA was extracted from all tissues of each sample using TRIzol (invitrogen, USA) and digested with DNase I (Waryong, China). Finally, the RNA was dissolved by adding 50 μl DEPC-treated water. After confirmed the integrity and quality, the total RNA was immediately stored at -80℃ for sequencing. The RNA samples with high purity (28S/18S ≥ 1.4) and high integrity (RIN ≥ 7.0) were employed for cDNA library construction.

Then, library construction and sequencing were performed by the Beijing Genomics Institute (BGI, China). Briefly, Oligo(dT)-attached magnetic beads were used to isolate mRNA. Purified mRNA was broken into short fragments by mixing with fragmentation buffer at appropriate temperature. The short fragments were purified and resolved with the Elution buffer for end repair and single nucleotide A addition, and then connected with adapters. Target fragments were selected as templates for PCR amplification to construct the cDNA sequencing libraries. Each cDNA library was sequenced by an Illumina HiSeq X-ten platform with paired end (PE) reads of 150 bp. The clean data were deposited in the NCBI (National Centre for Biotechnology Information) under the accession numbers PRJNA762437.

### Data filtering, mapping and gene expression profiling

To obtain high-quality clean reads, the adaptor sequences and the reads with unknown nucleotides larger than 5% or with low-quality nucleotides (≤ 10) larger than 20% were filtered out. Then the clean reads were mapped to the ginseng reference genome v1 (Ginseng Genome Database) using Bowtie2 (v2.2.5) [55], followed by gene expression levels of each sample were calculated using RSEM with default parameters, and the FPKM (Fragments Per Kilobase of transcript per Million mapped reads) value was used to quantify gene expression levels [56]. Pearson correlation coefficients (r^2^) of expression levels were calculated between each pair of ginseng samples using R package (v4.2.0), and samples with r^2^ < 0.5 across samples were removed. Differential expression analysis of each comparison group was performed according to the method described by DESeq2 (v1.16.1) [57]. The genes with a threshold of |log2(fold change)|≥ 1 and *p* ≤ 0.05 were identified as differentially expressed genes (DEGs).

### Weighted gene co-expression network construction

Weighted gene co-expression network analysis (WGCNA) was performed to construct co-expression networks of genes that were differentially expressed in different growth years of ginseng using the R package (v4.2.0) [58]. After background correction and quantile normalization, the all genes with top 50% variant in the variance analysis for WGCNA. Firstly, the hclust function was used to conduct cluster analysis on the expression data of the samples. Average was selected as the clustering method to remove the outlier samples. Then the function pickSoftThreshold was used to filter Soft Threshold (power). The similarity between the two genes was calculated by topological overlap (TOM), and the module gene tree was obtained by hierarchical clustering. The genes with the same expression patterns were grouped into the same module by dynamicTreeCut method, and the minimum number of genes was set as 30 in the module (minModuleSize = 30). The first principal component was used to calculate module eigengenes (ME) with mergeCutHeight = 0.25, and similar modules were merged through module characteristic genes. The cor (MEs, datTraits) function was used to estimate the module-trait relationship, then the Pearson correlation coefficient between the characteristic gene of the module and the growing years. The module with a correlation coefficient ≥| 0.8 | and *p* ≤ 0.05 was regarded as significant growing years-related modules. Finally, in order to further investigate these genes in significant related module, the enrichment analysis of these genes were analyzed based on Kyoto Encyclopedia of Genes and Genomes (KEGG) pathway by clusterProfiler (v3.16.1) [59]. Cytoscape (v3.7.0) was used to visualize the most significantly correlated genes with a WGCNA edge weight > 0.25, then we considered the top 20% of the connected genes as hub genes in the module [60]. The correlation between genes in the ginsenoside synthesis pathway and hub genes in the module was analyzed using R package (v4.2.0).

### Identification of differential expression genes (DEGs) associated with ginsenoside biosynthesis

Ginsenoside is the most important medicinal component in the ginseng. Therefore, the expression levels of genes involved in the biosynthesis of ginsenoside were analyzed. Then, DEGs associated with ginsenoside biosynthesis in each comparison group was also performed by DESeq2 (v1.16.1), which was consistent with the above [57].

### qRT-PCR validation

Fifteen DEGs were randomly selected for qRT-PCR to validate the RNA-seq data. Total RNAs as described for RNA-seq were used for qRT-PCR. Total RNA from each sample was reverse transcribed into cDNA using PrimeScript™ RT reagent Kit with gDNA Eraser (TaKaRa, Japan) following manufacturer’s protocol. Gene-specific primers were designed by Primer Premier 6 software [61]. The qRT-PCR reactions were performed in a final volume of 10 μl using 2 × SYBR Green qPCR Master Mix (Vazyme, China) on the Applied Biosystems® QuantStudio® 3 (Thermo Fisher Scientific, USA). PCR amplification was conducted under the following conditions: 95℃ for 5 min, 40 cycles of 95℃ for 30 s and 60℃ for 30 s, and with a dissociation stage of 95℃ for 15 s, 60℃ for 60 s and 95℃ for 15 s. Each biological replicate was technically replicated three times. The relative expression levels of the selected genes were calculated with the 2^−ΔΔCT^ method using GAPDH as the internal reference gene [62].

## Supplementary Information

**Additional file 1:** **TableS1.** Summary of the cultivated and wildginseng samples of different growth years. **FigureS1. **Theheatmap of Pearson correlation coefficients (PCCs) among biological replicatesof each sample. (A), YNJY; (B), JYSH; (C), TSBT; (D), PHQH; (E), YSWD. r^2^represents Pearson correlation value. R: root, S: stem, L, leaf. **FigureS2. **Determinationof the soft-thresholding power in the weighted gene co-expression networkanalysis (WGCNA) in the training set. (A), YNJY_vs_JYSH; (B), TSBT_vs_PHQH;(C), YONE_vs_YSWD. **Figure S3. **Co-expression networkanalysis across three groups. The correlation coefficients between differentmodules and traits are showed in a matrix. Each cell contains a correspondingcorrelation and *p*-value. (A), YNJY_vs_JYSH; (B), TSBT_vs_PHQH; (C),YONE_vs_YSWD. **FigureS4. **PCR verification on gene expression levels between RNA-seq analyses andqRT-PCR assays.

## Data Availability

The datasets generated and analyzed in the current study are available from the corresponding author on reasonable request. All data generated or analyzed during this study are included in this published article and its Supplementary information files. The clean RNA-seq data are freely available at: www.ncbi.nlm.nih.gov/bioproject/PRJNA762437.

## Abbreviations

PPD: Protopanaxadiol
PPT: Protopanaxatriol
IPP: Derivescisopentenyl pyrophosphate
DMAPP: Dimethylallyl pyrophosphate
MVA: Mevalonic acid
MEP: 2-C-methyl-D-erythritol-4-phosphate
GPS: Geranyl pyrophosphate synthase
FPS: Farnesyl pyrophosphate synthase
SS: Squalene synthase
SE: Squalene epoxidase
OSCs: Oxidosqualene cyclases
DS: Dammarenediol synthase
β-AS: β-Amyrin synthase
CYP450s: Cytochrome P450s
UGTs: UDP- glycosyltransferases
ME: Module eigengene
WGCNA: Weighted gene co-expression network analysis
FPS: Farnesyl diphosphate synthase
MVD: Diphosphomevalonate decarboxylase
PMK: Phosphomevalonate kinase
HMGR: Hydroxymethylglutaryl-CoA reductase
ISPG: (E)-4-hydroxy-3-methylbut-2-enyl-diphosphate synthase
ISPH: 4-Hydroxy-3-methylbut-2-en-1-yl diphosphate reductase
DXS: 1-Deoxy-D-xylulose-5-phosphate synthase
ISPF: 2-C-methyl-D-erythritol 2,4-cyclodiphosphate synthase
AACT: Acetyl-CoA C-acetyltransferase
HMGS: Hydroxymethylglutaryl-CoA synthase
ISPE: 4-Diphosphocytidyl-2-C-methyl-D-erythritol kinase
MVK: Mevalonate Kinase
DXR: 1-Deoxy-D-xylulose-5-phosphate reductoisomerase
KEGG: Kyoto Encyclopedia of Genes and Genomes
FPKM: Fragments Per Kilobase of transcript per Million mapped reads
