# Supplementary material for: Effects of growth years on ginsenoside biosynthesis of wild ginseng and cultivated ginseng
Source: BMC Genomics. 2022 Apr 23;23:325. doi: 10.1186/s12864-022-08570-0 (PMC9035264; doi:10.1186/s12864-022-08570-0)
Supplement: Supplementary file 1 — Additional file 1: TableS1. Summary of the cultivated and wildginseng samples of different growth years. FigureS1. Theheatmap of Pearson correlation coefficients (PCCs) among biological replicatesof each sample. (A), YNJY; (B), JYSH; (C), TSBT; (D), PHQH; (E), YSWD. r2represents Pearson correlation value. R: root, S: stem, L, leaf. FigureS2. Determinationof the soft-thresholding power in the weighted gene co-expression networkanalysis (WGCNA) in the training set. (A), YNJY_vs_JYSH; (B), TSBT_vs_PHQH;(C), YONE_vs_YSWD. Figure S3. Co-expression networkanalysis across three groups. The correlation coefficients between differentmodules and traits are showed in a matrix. Each cell contains a correspondingcorrelation and p-value. (A), YNJY_vs_JYSH; (B), TSBT_vs_PHQH; (C),YONE_vs_YSWD. FigureS4. PCR verification on gene expression levels between RNA-seq analyses andqRT-PCR assays. [file 12864_2022_8570_MOESM1_ESM.docx]

Effects of growth years on ginsenoside biosynthesis of wild ginseng and cultivated ginseng

Xiaoxue Fang^1^, Manqi Wang^1^, Xinteng Zhou^1^, Huan Wang^1^, Huaying Wang^1*^, Hongxing Xiao^1*^

^1^ Key Laboratory of Molecular Epigenetics of Ministry of Education, Northeast Normal University, Changchun 130024, China

*** Correspondence:**

Huaying Wang

wanghx609@nenu.edu.cn

Hongxing Xiao

xiaohx771@nenu.edu.cn

**Table S1.** Summary of the cultivated and wild ginseng samples of different growth years.

|  | Groups | Locations | Growing years | Tissue | Numbers |
| --- | --- | --- | --- | --- | --- |
| cultivated ginseng  cultivated ginseng | YNJY_R | Jingyu county,  Jilin Province | 1  1  1 | root | 2 |
|  | YNJY_S |  |  | stem | 3 |
|  | YNJY_L |  |  | leaf | 3 |
|  | JYSH_R |  | 6  6  6 | root | 2 |
|  | JYSH_S |  |  | stem | 2 |
|  | JYSH_L |  |  | leaf | 2 |
|  | TSBT_R | Taishang Town, Jilin Province | 6  6  6 | root | 2 |
|  | TSBT_S |  |  | stem | 3 |
|  | TSBT_L |  |  | leaf | 3 |
|  | PHQH_R |  | >15  >15  >15 | root | 2 |
|  | PHQH_S |  |  | stem | 3 |
|  | PHQH_L |  |  | leaf | 3 |
| wild ginseng | YONE_R | Korean Autonomous County of Changbai,  Jilin Province | 1  1  1 | root | 1 |
|  | YONE_S |  |  | stem | 1 |
|  | YONE_L |  |  | leaf | 1 |
|  | YSWD_R |  | 20  20  20 | root | 2 |
|  | YSWD_S |  |  | stem | 2 |
|  | YSWD_L |  |  | leaf | 2 |


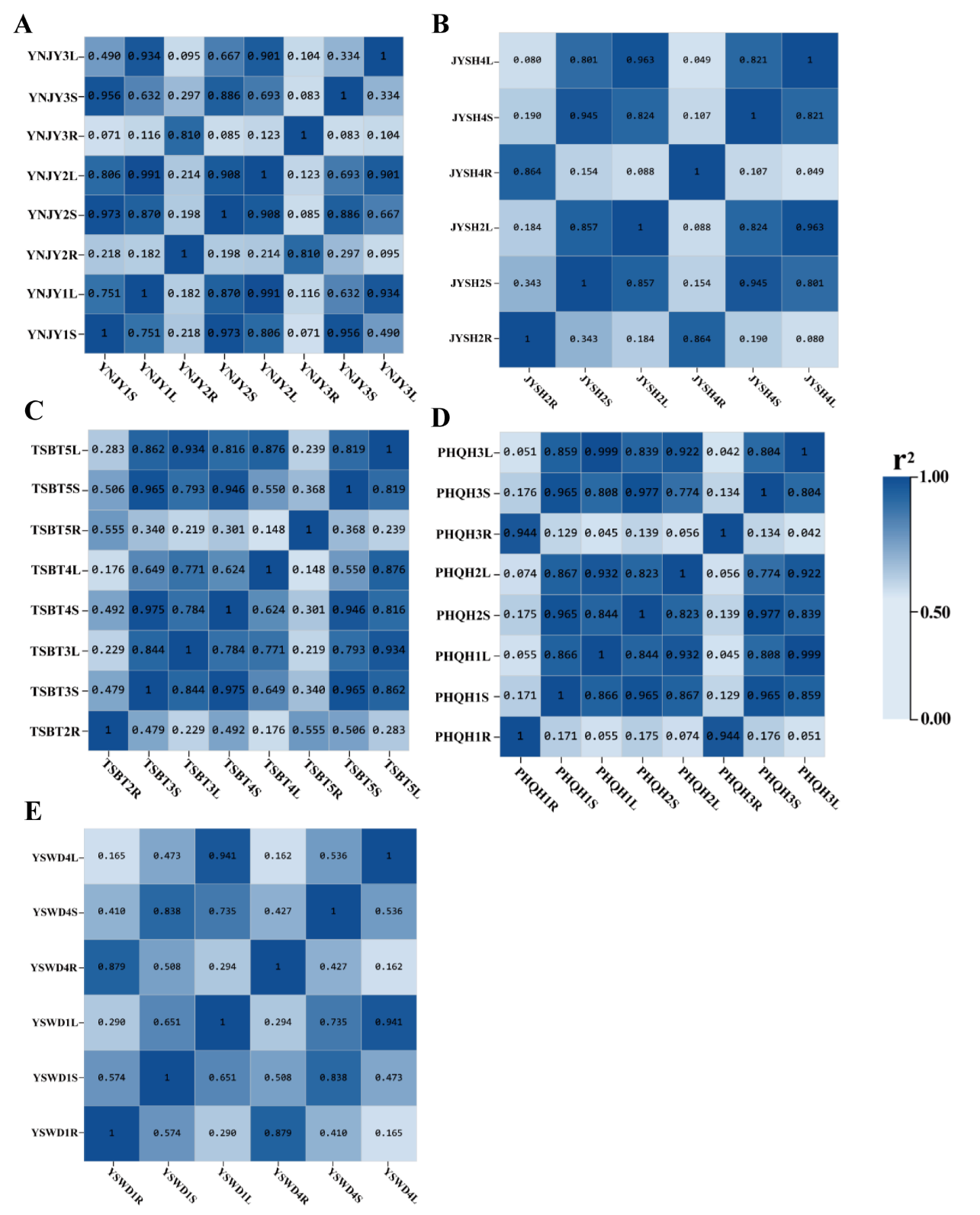


**Figure S1.** The heatmap of Pearson correlation coefficients (PCCs) among biological replicates of each sample. (A), YNJY; (B), JYSH; (C), TSBT; (D), PHQH; (E), YSWD. r^2^ represents Pearson correlation value. R: root, S: stem, L, leaf.


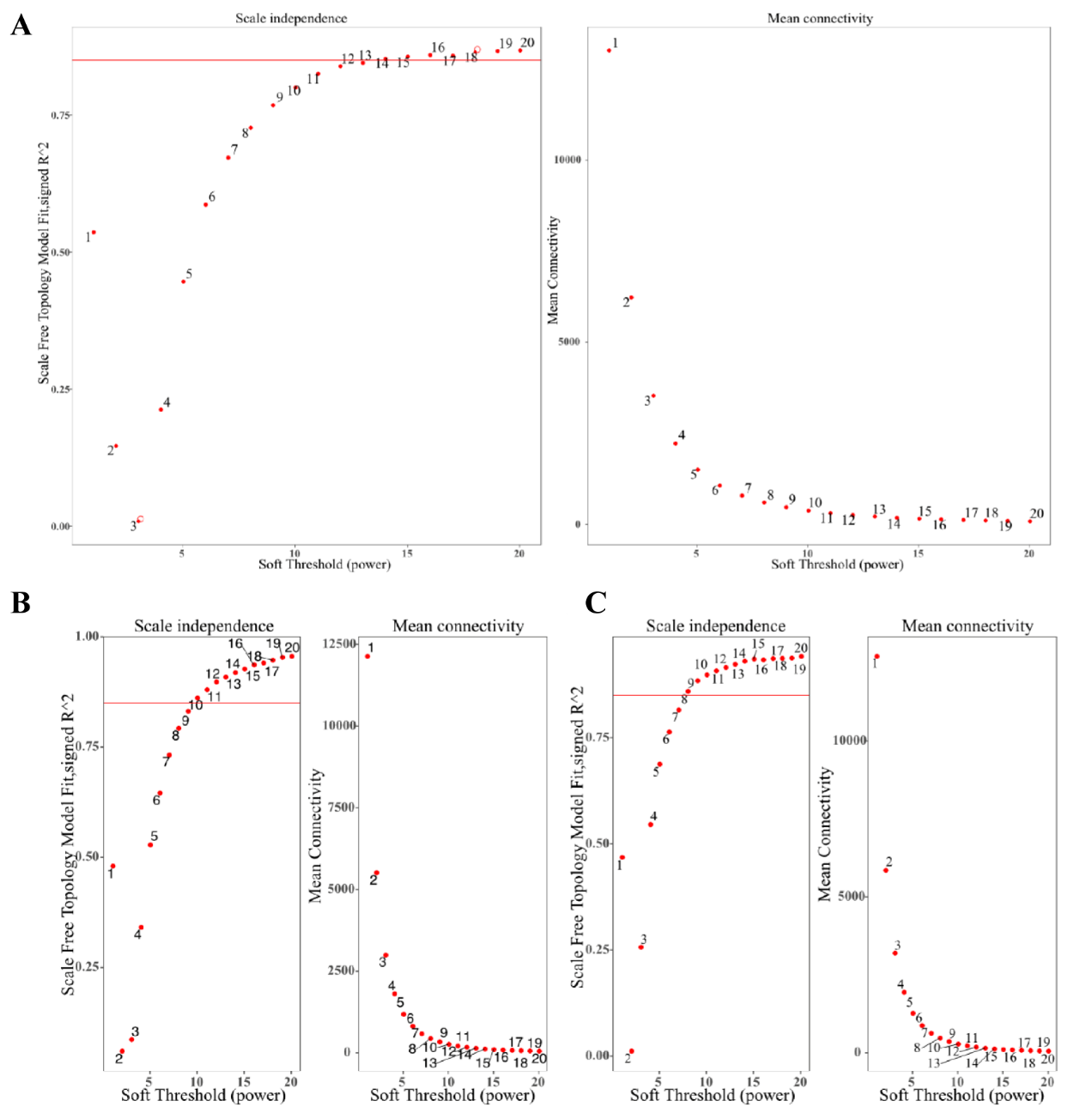


**Figure S2.** Determination of the soft-thresholding power in the weighted gene co-expression network analysis (WGCNA) in the training set. (A), YNJY_vs_JYSH; (B), TSBT_vs_PHQH; (C), YONE_vs_YSWD.


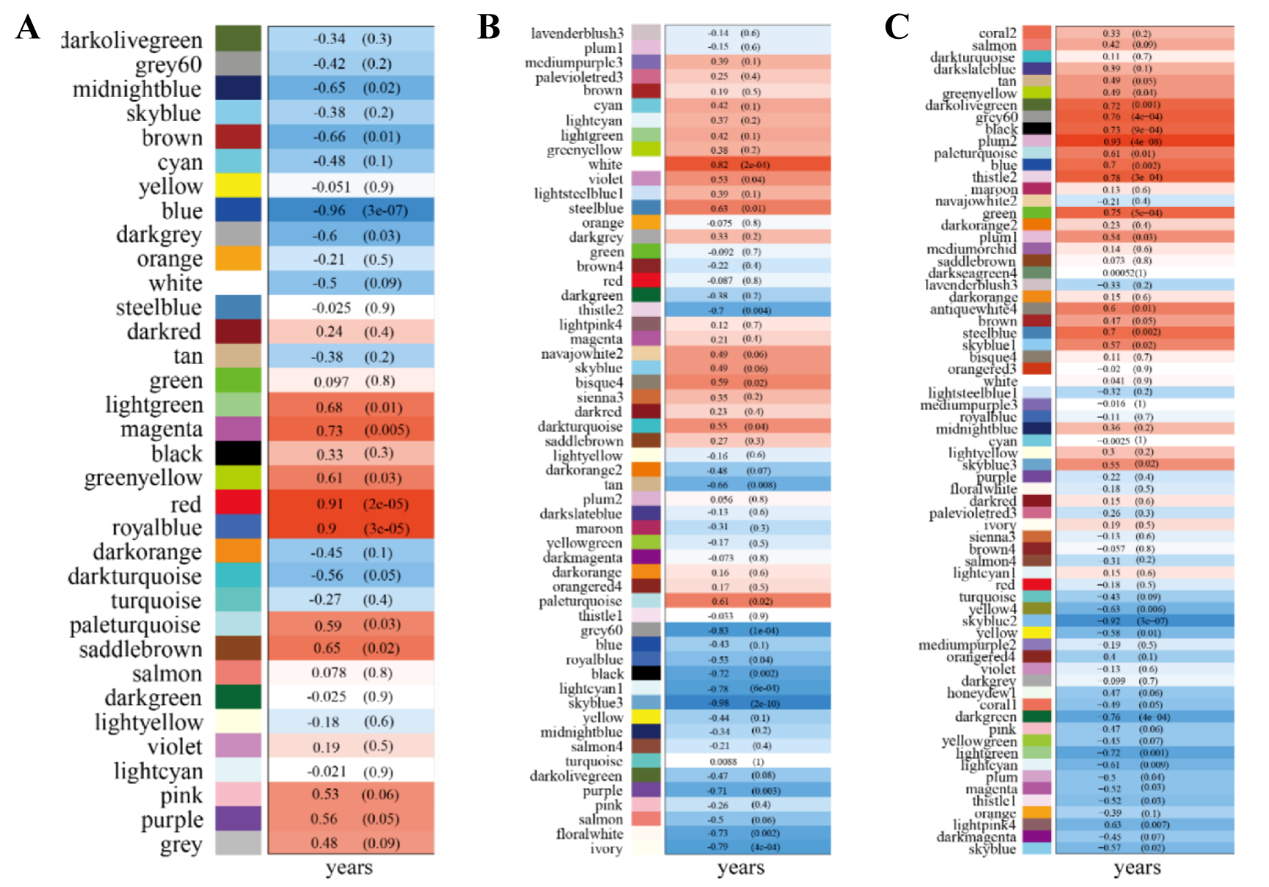
**Figure S3.** Co-expression network analysis across three groups. The correlation coefficients between different modules and traits are showed in a matrix. Each cell contains a corresponding correlation and p-value. (A), YNJY_vs_JYSH; (B), TSBT_vs_PHQH; (C), YONE_vs_YSWD.

**Figure S4.** PCR verification on gene expression levels between RNA-seq analyses and qRT-PCR assays.
